# The impact of nursing practice environments on patient safety culture in primary health care: a scoping review

**DOI:** 10.3399/BJGPO.2023.0062

**Published:** 2024-01-10

**Authors:** Soraia Cristina de Abreu Pereira, Olga Maria Pimenta Lopes Ribeiro, Cíntia Silva Fassarella, Eduardo José Ferreira Santos

**Affiliations:** 1 PhD student of Nursing Science at Abel Salazar Biomedical Sciences Institute, University of Porto, Porto, Portugal, Invited Adjunct Professor at Red Cross Northern School of Health and CINTESIS@RISE, Porto University, Porto, Portugal; 2 Adjunct Professor at Nursing School of Porto and CINTESIS@RISE, Porto University, Porto, Portugal; 3 Adjunct Coordinator and Permanent Professor of the Graduate Program in Nursing, Adjunct Professor at the Faculty of Nursing of the State University of Rio de Janeiro, Rio de Janeiro, Brazil; 4 Adjunct Professor at Polytechnic University of Viseu, School of Health; Health Sciences Research Unit: Nursing (UICISA: E) and Portugal Centre for Evidence Based Practice (PCEBP): a JBI Centre of Excellence, Coimbra, Portugal

**Keywords:** nursing, work environment, patient safety, primary health care

## Abstract

**Background:**

Patient safety has in recent decades become a global concern. It is a key priority area of healthcare organisations, and has a direct impact on patient health and wellbeing. Work environments can strongly impact nurses' wellbeing and may ultimately produce different outcomes for both professionals and patients. The adverse events occurrence is an example of how work environments influence outcomes, and there is evidence of this correlation in several studies conducted in recent years.

**Aim:**

To map the knowledge regarding the impact that nursing practice environments have on safety culture in primary healthcare settings, as primary health care concentrates a significant portion of the population’s care.

**Design & setting:**

This review was conducted following the methodology proposed by the Joanna Briggs Institute (JBI) for scoping reviews.

**Method:**

Study selection, data extraction, and synthesis were performed by two independent reviewers. Based on Population (or participants), Concept, and Context (PCC) framework, studies were considered that addressed nurses' practice environment and patient safety culture in primary health care. All studies published or unpublished from 2002 to the present were considered.

**Results:**

Seven studies were included in this review; however, the existing evidence on the relation between nurses' practice environments and patient safety is still limited in primary healthcare settings. Although clear evidence was not found, several characteristics of nursing practice environments that may impact healthcare safety were found, such as leadership, communication, and organisational culture and policies.

**Conclusion:**

More research directed at primary healthcare nursing practice settings is needed and could be valuable in defining and implementing strategies that promote the safety of care.

## How this fits in

Work environments strongly influence the physical, psychological, and emotional wellbeing of professionals, and are responsible for several outcomes for both professionals and patients. Patient safety and the occurrence of adverse events is an example of this, and there is evidence of this relationship in several studies conducted in recent years. However, many of the studies were conducted in hospital settings, with an apparent undervaluation of primary health care. It is essential to understand the impact that nursing practice environments have on patient safety culture in the specific context of primary health care. This knowledge is fundamental not only for a more effective and adequate understanding of this phenomenon but also to enable the definition of strategies promoting safe care.

## Introduction

Patient safety has become a global concern in the past few years and is a key area of health organisations, with direct impacts on patient health and wellbeing.^1^ Patient safety is defined by the World Health Organization (WHO) as *'the reduction of risk of unnecessary harm associated with health care to an acceptable minimum* ^2^' and safety culture is considered the *'product of individual and group values, attitudes, perceptions competencies, and patterns of behaviour that determine the commitment to, and the style and proficiency of, an organisation’s health and safety management'.* ^2^ Unsafe care is one of the leading causes of disability and death, worldwide, according to data from the WHO, raising the need for tailored interventions to promote safe care.^3^ Higher rates of unsafe care and the occurrence of adverse events and errors have been linked to unfavourable work environments and poor working conditions.^4^ The International Council of Nurses (ICN) believes that a safe work environment, appropriate compensation, professional recognition and appreciation, adequate material resources that are tailored to meet needs, and human resource policies that are focused on the recruitment and retention of professionals are all essential components of a favourable work environment.^5^

In the nursing field, there have been several reports and initiatives worldwide that have drawn attention to the importance of nurses' working environments as a catalyst for healthcare quality,^3,6^ such as the 2004 report *Keeping Patients Safe: Transforming the Work Environment of Nurses* ^7^ from the Institute of Medicine (IOM), or the World Patient Safety Day of 2020, which established the theme *'Safe health workers, safe patients'* as a strategy to evidence the connection between patient safety and professional practice environments.^3,8^

Recent research on the relationship between work environments and job satisfaction, burnout, omitted care, and the intention to leave the profession has led to a better understanding of this relationship.^9,10^ Work environments have a significant impact on professionals' wellbeing and contribute to a variety of outcomes for professionals as well as patients.^8^ Heavy workloads, shortage of human and material resources, lack of communication and teamwork,^4^ or even low involvement of professionals in the definition of organisational policies and decision-making increase the risk of adverse events^8^ and reduce patient safety-related scores.^1^

Because of its comprehensive nature, primary health care focuses a significant portion of the population’s care.^9–13^ Primary health care is understood by the WHO as *'a whole-of-society approach to health that aims at ensuring the highest possible level of health and wellbeing and their equitable distribution by focusing on people’s needs and as early as possible along the continuum from health promotion and disease prevention to treatment, rehabilitation and palliative care, and as close as feasible to people’s everyday environment'.* ^14^ Primary health care includes services for health promotion; disease prevention; community promotion and development; and curative, rehabilitative, and palliative care, and constitutes first-level, appropriate, and evidence-based care.^13,14^

A large number of studies have been conducted mainly in hospitals, with an apparent undervaluation of primary health care,^15–18^ although some research has recently begun to emerge in this context.^17–23^

According to Kuriakose *et al*,^17,24^ about 20%–25% of the population experience errors or adverse events in primary health care, with diagnostic errors, communication gaps, unsafe medication practices, and fragmentation of care appearing as contributing factors to unsafe care. Interactions between patients and professionals are generally limited, may occur at intervals of weeks to months, and in some cases these interactions happen in the patient’s home.^16,17^ Safety incidents usually occur outside office hours or outside the healthcare setting, in contrast to the hospital environment where the patient is under supervision 24 hours a day, which may contribute to the under-reporting of errors or incidents and to an idea of low risk of incidents.^15–17^ Lack of access to patient history, insufficient medical knowledge, high workloads, an ageing population, and the increase in chronic and complex diseases are among the common causes of these incidents.^17,18^

In the particular context of primary health care, it is essential to understand the influence that nursing practice environments have on patient safety culture, with a substantial proportion of health care provided in this setting, and research that has suggested that patients are also at risk of errors and adverse events.^15–17^

To understand whether there were studies on the topic, a preliminary search was conducted in JBI Evidence Synthesis, Cochrane Database of Systematic Reviews, PROSPERO, Open Science Framework (OSF), and MEDLINE. Following this search, two scoping reviews were identified and targeted for the analysis. One of the reviews aimed to map the evidence related to nursing practice settings in primary health care; however, it did not refer to patient safety.^22^ The other review aimed to identify the challenges to patient safety in primary health care but did not address the issue of nursing practice settings.^23^ No other current or in-progress systematic reviews on the topic were found.

Thus, and given that the primary evidence identified is still poorly described, this review was conducted according to a methodology proposed by JBI,^25^ to map the knowledge about the impact that professional nursing practice environments have on the culture of safety in primary healthcare settings. Considering the above, the review question was: 'what impact do nursing professional practice environments have on patient safety culture in primary healthcare settings?'

## Method

This review was performed according to the methodology proposed by JBI for scoping reviews^26^ and the Preferred Reporting Items for Systematic Reviews and Meta-Analyses for Scoping Reviews (PRISMA-ScR)^27^ were followed when writing this review. A protocol for this review was developed and has been published previously and provides details of all the steps performed.^28^

### Eligibility criteria

The PCC framework was followed to define the eligibility criteria.^29^ Table 1 states the criteria and PCC definitions followed in this review.

**Table 1.** Eligibility criteria and definitions

Category	Criteria
Population	Studies that included nurses in any field of action.
Concept	Studies that mapped evidence related to patient safety culture and nurses' professional practice environments.Studies that guided or described nurses' professional practice environments, and that also considered suggestions related to patient safety or patient safety culture.Studies that included and related these two concepts, with the following definitions:Patient safety: *'The reduction of risk of unnecessary harm associated with health care to an acceptable minimum.'* ^3^Safety culture: *'Product of individual and group values, attitudes, perceptions competencies, and patterns of behaviour that determine the commitment to, and the style and proficiency of, an organisation’s health and safety management'.* ^3^Nursing work environment: *'The set of characteristics of the work context that facilitate or constrain the professional nursing practice.'* ^56^
Context	Studies conducted in primary care settings, primary healthcare organisations or centres and community services or centres, regardless of country of origin or socio-cultural environment.Studies that included the context of primary health care, with the following definition:Primary health care: *'is a whole-of-society approach to health that aims at ensuring the highest possible level of health and wellbeing and their equitable distribution by focusing on people’s needs and as early as possible along the continuum from health promotion and disease prevention to treatment, rehabilitation, and palliative care, and as close as feasible to people’s everyday environment.*"^13^

### Search strategy

A research strategy consisting of three phases was used. Initially, a limited search was conducted in MEDLINE (PubMed) and CINAHL (Cumulative Index to Nursing and Allied Health Literature; EBSCO) to identify articles on the topic under analysis. The text words contained in the titles and abstracts of the articles considered relevant were consulted and the index terms were used to develop a full search strategy, available in Table 2.

**Table 2.** Database search strategy and results

Database: Medline (PubMed) Filters: 2002-present Results: 234 Search strategy (January 25, 2023) (("nursing"[MeSH Terms] OR "nurs*"[Title/Abstract] OR "nursing staff"[MeSH Terms] OR "nurses"[MeSH Terms] OR "nursing practice"[Title/Abstract]) AND ("workplace"[MeSH Terms] OR "work environment"[Title/Abstract] OR "working conditions"[MeSH Terms] OR "work setting"[Title/Abstract] OR "professional practice"[MeSH Terms] OR "practice environment"[Title/Abstract] OR "positive work environment"[Title/Abstract]) AND ("patient safety"[MeSH Terms] OR "patient safet*"[Title/Abstract] OR "patient harm"[MeSH Terms] OR "patient harm"[Title/Abstract] OR "safety management"[MeSH Terms] OR "patient risk*"[Title/Abstract] OR "safety"[MeSH Terms] OR "safety culture"[Title/Abstract] OR "safety climate"[Title/Abstract] OR "patient safety culture"[Title/Abstract] OR "medical errors"[MeSH Terms] OR "adverse event"[Title/Abstract] OR "patient outcomes"[Title/Abstract]) AND ("primary health care"[MeSH Terms] OR "primary care"[Title/Abstract] OR "primary health care"[Title/Abstract] OR "primary healthcare"[Title/Abstract] OR "community health servic*"[Title/Abstract] OR "community health centers"[MeSH Terms] OR "community health center*"[Title/Abstract])))
Database: CINAHL Complete (EBSCO) Filters: 2002-01-01 – 2023-12-31 Results: 53 Search strategy (February 13, 2023) (AB (nurs* OR nursing practice) OR (MH "nursing care") OR (MH "staff nurses") OR (MM "nurses”)) AND (AB (work environment OR working conditions OR work setting OR practice environment OR positive work environment) OR (MM "work environment") OR (MH "professional practice")) AND (AB (patient safet* OR patient harm OR safety management OR patient risk OR safety culture OR safety climate OR patient safety culture OR adverse event OR patient outcomes) OR ("MH patient safety”) OR (MH "safety") OR (MH "cultural safety") OR (MM "health care errors") OR (MM “adverse health care event")) AND (AB (primary care OR primary health care OR primary healthcare OR community health servic* OR community health center) OR (MH "primary health care") OR (MH "community health centers") OR (MM "Community Health Nursing"))
Database: Embase (Elsevier) Results: 419 Search strategy (February 10, 2023) (('nursing'/exp OR nurs*:ab,ti OR nursing) AND practice:ab,ti AND (((((((((((((((('workplace'/exp OR work) AND environment:ab,ti OR work) AND 'environment'/exp OR work) AND setting:ab,ti OR professional) AND 'practice'/exp OR practice) AND environment:ab,ti OR positive) AND work AND environment:ab,ti AND patient AND 'safety'/exp OR patient) AND safet*:ab,ti OR patient) AND harm OR patient) AND harm:ab,ti OR patient) AND risk*:ab,ti OR 'safety'/exp OR safety) AND culture:ab,ti OR safety) AND climate:ab,ti OR patient) AND safety AND culture:ab,ti OR medical) AND 'error'/exp OR adverse) AND event:ab,ti OR patient) AND outcomes:ab,ti AND ((((((primary AND health AND 'care'/exp OR primary) AND care:ab,ti OR primary) AND health AND care:ab,ti OR primary) AND healthcare:ab,ti OR community) AND health AND servic*:ab,ti OR health) AND 'center'/exp OR community) AND health AND center*:ab,ti) AND (2002:py OR 2004:py OR 2005:py OR 2006:py OR 2007:py OR 2008:py OR 2009:py OR 2010:py OR 2011:py OR 2012:py OR 2013:py OR 2014:py OR 2015:py OR 2016:py OR 2017:py OR 2018:py OR 2019:py OR 2020:py OR 2021:py OR 2022:py OR 2023:py)
Database: RCAAP – Repositório Científico de Acesso Aberto de Portugal Filters: 2002-01-01 – 2023-12-31 Results: 5 Search strategy (February 02, 2023) (Patient safety [description]) AND (Nurs* [description]) AND (Work environment [description]) AND (Primary health care [description])
Database: WHO – World Health Organization database Filters: 2002-01-01 – 2023-12-31 Filters: WHO Publications – Health Topic: Patient safety Results: 72 Search strategy (February 06, 2023)
Database: Agency for Health Research and Quality database Filters: Setting of care: ambulatory care; Clinical area: nursing; Data range: 01-01-2002 – 12-31-2023 Results: 6 Search strategy (February 02, 2023) “patient safety” AND “environment” AND “nursing”
Database: WorldCat Filters: 2002–2023 Results: 9 Search strategy (February 02, 2023) kw:"nurs*" AND kw: "work environment" AND kw:"patient safety" AND kw:"primary health care"
Database: ProQuest Dissertations and Theses Filters: Data Range: 2002–2023; Source type: Dissertations and Theses Results: 11 Search strategy (February 02, 2023) AB("patient safety") AND AB(primary care) AND AB("work environment")

* = truncation.

Subsequently, and using all the keywords and index terms initially identified, a second search was conducted in the following databases: MEDLINE (PubMed); CINAHL (EBSCO); Embase (Elsevier); Repositório Científico de Acesso Aberto de Portugal (RCAAP); WHO; Agency for Health Research and Quality; WorldCat; and ProQuest Dissertations and Theses Global. Published and unpublished literature in any language from 2002 to the present was considered, as the WHO Executive Board extensively discussed the topic of patient safety in 2002, and since then many initiatives have taken place at a global level, making it an important milestone. In the third phase of the search, the reference lists of the studies included in this review were scanned, but no additional relevant studies were found.

### Study selection

**Figure 1.**
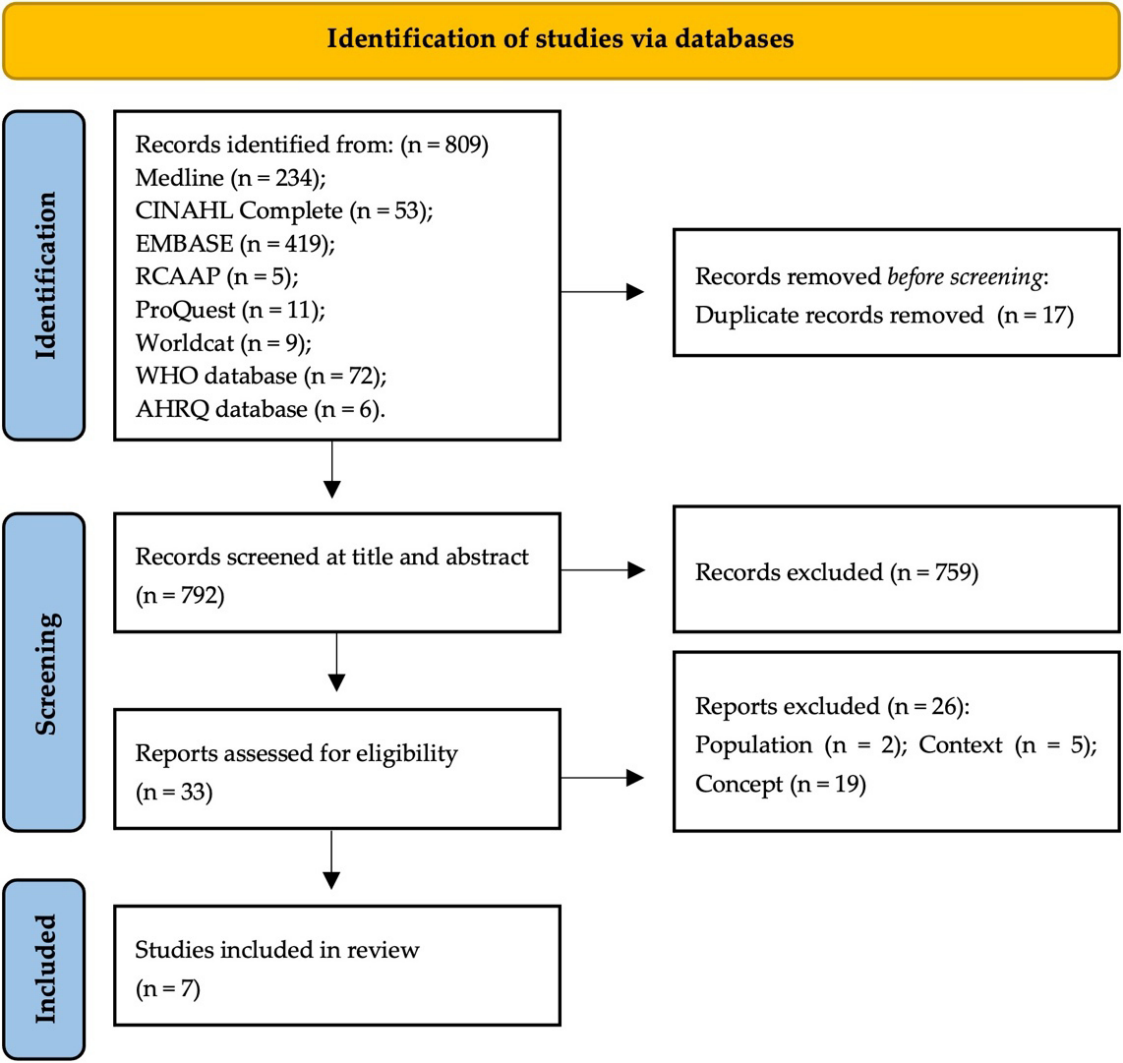
PRISMA-ScR flowchart. AHRQ = Agency for Health Research and Quality. RCAAP = Repositório Científico de Acesso Aberto de Portugal. WHO = World Health Organization.

All studies identified in the search were collected and integrated into EndNote (version X9.3.3), duplicates were removed, and citations were imported into Rayyan. Two independent reviewers (SP and OR) scanned the titles and abstracts to assess the previously defined inclusion criteria. A pilot review process of initial titles and abstracts was conducted independently by both reviewers and with over 75% agreement between the reviewers, it was decided to make no changes to the eligibility criteria. After the assessment of all titles and abstracts, the studies that fulfilled the inclusion criteria were read in integral form. Disagreements between the reviewers were resolved by constructive discussion and a consensus was reached without the need for a third reviewer (ES). The full results of the search and the reasons for the exclusion of studies after reading the full text were recorded and are presented in a PRISMA flowchart^30^ (Figure 1).

### Data extraction

Data were extracted from selected studies by two independent reviewers (SP and OR) using a data extraction tool developed by the reviewers’ team. The data extracted included specific details about the concept, context, study methods, and specific objectives. The authors of the included articles were not contacted for further information or data clarification because there was no need.

### Data analysis and presentation

The extracted data have been presented in tabular form and a narrative synthesis accompanies the results, describing how the results relate to the purpose and to the question formulated for this review. Additionally, the narrative synthesis was based on a hermeneutic analysis of the phenomenon, which followed the principles proposed by Bardin.^31^

## Results

A total of 809 studies were retrieved from the databases and, from those, 792 remained after removing duplicates. After the analysis phases of the review process, seven studies met the inclusion criteria and were included in this review, having their characteristics, objectives, and main results detailed in Table 3.

**Table 3.** Summary of the included studies

Author(s), year, country	Aims	Type of study	Results
Burke, 2013, UK^42^	To highlight the issue of work-related stress in the work environment of community nurses	Literature review	Inability to finish work within working hours is the primary source of stress for community nurses. The main obstacle that has been identified is the workload and the shortage of human resources. Nurses' performance is affected when they don't get enough time to rest, which can compromise patient and professional safety.
Poghosyan *et al*,2016, US^40^	To propose a comprehensive model for maximising nurse practitioner contributions to primary care, which includes the factors affecting nurses' care and patient outcomes and explains their interrelated impact	Literature review	Medical errors, accidents, and unsafe work have all been linked to unfavourable practice environments marked by poor professional relationships, ineffective communication, issues with leadership, and a lack of support and resources.Nurses may be predisposed to job dissatisfaction, burnout, and a negative impact on the safety and quality of care owing to the complexity of patients, heavy workloads, and constantly shifting organisational structures.
Gabrani *et al*,2016, Albania^32^	To determine the safety attitudes of specialist physicians, general physicians, and nurses in primary care in Albania	Cross-sectional study	Nurses' positive responses to the safety climate and teamwork were lower compared to other professionals such as specialist physicians and general physicians. The results showed a positive response regarding teamwork, job satisfaction, perceived leadership, communication, stress recognition, and safety climate by the nurses.
Nejati *et al*, 2016, US^44^	To understand the main causes of nurses' fatigue, the barriers that prevent nurses from taking restorative breaks, and the consequences of nurse fatigue for staff, patient, and facility outcomes	Systematic literature review	The quality of life of nurses is negatively impacted by fatigue, which can also hinder their performance and raise the likelihood of medical errors. Nurses' wellbeing can suffer because of physical and mental health issues brought on by long shifts, insufficient sleep, and a lack of rest breaks.
Souza, 2017, Brazil^35^	To evaluate the patient safety culture in the primary health care of a city in the central-western region of the State of Rio Grande do Sul, Brazil	Cross-sectional study(Master’s dissertation)	Participants in the study evaluated safety culture negatively. Nearly all domains studied showed negative values for safety culture. Nurses showed poorer results in the domain of 'job satisfaction' than other health professionals. The domains 'working conditions' and 'error' were the worst scored.
Mesquita, 2017,Brazil^34^	To investigate the safety patient culture in the nursing professionals' perspective of the primary health care	Exploratorydescriptive qualitativestudy(Master’s dissertation)	The findings point to a negative patient safety culture because of the weaknesses that were found in several dimensions.Knowledge, continuing education, risk prevention, team commitment to patient safety, punitive error culture, teamwork, job satisfaction, patient and family involvement, and the use of protocols and the best scientific evidence were all found to be lacking in the patient safety culture.
Rogers, 2021, UK^43^	To explore possible strategies to promote the health and wellbeing of community nurses and the barriers they meet	Literature review	High levels of employee turnover and intentions to leave are a result of working in stressful environments, affecting patient outcomes and safety. Stress that is not properly controlled can result in compassion fatigue, which is linked to an increase in nursing errors.

Evidence on the relationship between nurses' practice environments and patient safety is still limited in primary healthcare settings. There has been a large investment in patient safety; however, most researchers and investigations were conducted only in hospital settings.^32^ Regarding the assessment of safety culture, the cross-sectional studies that were included in this review show that the overall assessment of patient safety culture is negative.^33,34^ There are also reports of a lack of knowledge about patient safety,^23^ and resigned attitudes towards adverse events and errors,^32^ with a general perception that the primary healthcare environment is susceptible to the occurrence of adverse events and errors.^32–35^ Several of the protective factors and factors considered barriers to promoting patient safety found in this review (Table 4) are elements that characterise nursing practice environments, so it can be interpreted that there is a clear relationship between these two concepts.^32,34,36–38^

**Table 4.** Protective factors and barriers to promoting patient safety

Protective factors	Barriers
Knowledge about patient safety and ongoing training; existence of risk and incident prevention systems; commitment to patient safety; teamwork; job satisfaction; evidence-based clinical practice; patient and family involvement in care.	Professionals' difficulty in discussing errors; lack of or compromised communication within the multidisciplinary team; lack of or compromised communication with the patient and family; punitive culture towards errors.

A content analysis of the articles included in this review was performed, and the following seven categories emerged: collaborative work; leadership; workload and nurses' health; recognition and valorisation of nurses; organisational policies; professional development opportunities; and strategies to promote patient safety and positive nursing practice environments.

### Collaborative work

Relationships among nurses and between nurses with other professionals in the multidisciplinary team, clinical support, collaborative decisionmaking, and communication were found to be all relevant aspects of teamwork that characterised nursing practice environments.^36,39^

The relationships between nurses and the multidisciplinary team played an important role in promoting constructive and safer practice environments. In the literature review conducted by Poghosyan *et al*,^40^ it was found that favourable relationships between nurses and physicians, including effective communication, knowledge sharing, and teamwork, facilitated the development of a positive practice environment. The relationships between nurses and administrative staff were also highlighted, with administrative support and the promotion and respect for the role and activities developed by nurses and administrative staff, respectively, being particularly important.^40^

Ineffective communication between nurses and the rest of the team included a lack of respect and collegiality or insufficient support and resources for nursing practice.^32^ Ineffective communication or lack of communication was considered a barrier to safe care and was associated with errors, accidents, unsafe work behaviours, and other adverse outcomes.^18,32,33,35,40,41^ Communication was even pointed out as an essential skill and strategy that should be used by primary healthcare providers to improve patient safety.^32^

### Leadership

Nurse managers should be encouraged and have leadership skills and commitment to patient safety and nurse wellbeing.^32,34,42,43^ In the two cross-sectional studies included in this review, management was perceived as positive.^32,34^ Staff wellness and supportive nursing practice environments were associated with better quality care delivery and lower occurrence of adverse events.^32,34,42,43^ It was also found that nurse managers must be properly empowered to provide nurses with a work environment that enhances their wellbeing and the wellbeing of the team.^39,40,42,43^ Access to human and material resources, counselling the team, clinical support and backup, emotional stress management skills, and the ability to identify situations of compassion fatigue and burnout were areas that the nurse manager must know how to manage to care for the team and promote quality of care.^39,42,43^

The development of resilience in nurses was also considered an essential point not only for promoting wellbeing but also for ensuring quality care.^40,43^ The nurse manager must be able to recognise nurses' difficulties, and promote guidance and training in emotional resilience and emotional intelligence, supporting the team with compassion so that they can function effectively.^40,43^ Also here, nursing practice environments must be positive and constructive, allowing nurses to address the problems they face in an open and fair way.^40,43^

### Workload and nurses' health

The primary healthcare nursing practice environment was found to be a unique scenario with a wide variety of sources of stress.^39,42^ The increasing amount of work and the gap between the workload and the ability of professionals to cope with their needs led to many professionals feeling exhausted and experiencing stress-related illnesses.^40,43^ Professionals increasingly felt that they were unable to complete work at scheduled times, they needed to take work home, and they needed to work during their lunch break.^39,42,44^

The consequence of this lack of break moments during the workday was documented in several studies, concluding that the increasing workloads associated with the shortage of professionals are identified as one of the main barriers to continuing to work as a nurse.^40,42,43,45–47^ Working >40 hours per week without breaks caused staff functioning to decline, which could result in safety issues for patients and the staff themselves,^39,40,42,43,48^ as well as a considerably increased likelihood of error and an increased risk of adverse events.^43^

Concrete evidence was found that working under extreme circumstances was the main factor causing workplace stress, higher levels of staff turnover, and intentions to leave the profession, which produced consequences on nurses' wellbeing, making them more likely to suffer from burnout and compassion fatigue.^40,43,44,48–51^

Fatigue had a negative impact on nurses' quality of life, and was widely associated with long working hours, consecutive shifts, insufficient hours of sleep, long travel or walking, and lack of rest breaks.^42,44,48^ Staff fatigue could affect the quality of care provided, which may be translated into institutional outcomes and associated costs.^42,44^ Several negative aspects related to fatigue were described, such as fatigue owing to sleep deficiency that reduced performance on psychomotor vigilance tasks, increased frequency, and duration of attention lapses, slowed response times, created errors of omission, or impaired problem-solving ability.^42,44^ The association of errors in health care and sleep fatigue was also described as an important predictor of errors, with the incidence of errors increasing when nurses' hours of regular sleep decreased.^42,44,48^ Error rates and the number of hours nurses worked in a row were also mentioned. Nurses who worked ≥12.5 hours in a row were three times more likely to make mistakes than nurses who worked shorter shifts.^42–44,48^

### Recognition and valorisation of nurses

Recognising and promoting the visibility of the nurse’s role within the healthcare organisation was important and was related to favourable nursing practice environments.^40,41^ Nurses often experienced low visibility regarding their role and felt they received less support than other medical professionals,^40,41^ which contributed to their job dissatisfaction and was related to a higher risk of errors.^42,44^

It is understood that job satisfaction, the increasing complexity of community nursing work, and high stress levels are associated with job retention problems, which have become a problem globally.^40^ Stress management should be a priority to produce feelings of appreciation among nurses.^40^ Recognising nurses' professional autonomy and promoting feelings of belonging and commitment were also essential to ensure nurses' wellbeing, and for them to feel supported and motivated.^40^

Although COVID-19 brought some recognition for nurses working in hospital settings, it is reported that for nurses working in primary health care, there was little support and recognition, despite the increased workload and the need to maintain levels of care delivery.^40,43,52^ Although nurses are key players in healthcare systems, they often felt invisible and unappreciated,^40,43^ which could be translated into a risk to patient safety and a higher risk of adverse events.^53^

### Organisational policies

Policies, regulations, and the influence of organisations had a considerable role in nurses' performance and quality of care.^40,41^ Institutional barriers to promoting positive nursing practice environments, such as lack of staff rest areas, denying nurses breaks, high-workload assignments, insufficient hiring of human resources, and inadequate management of human and material resources, were examples of institutional policies that undermined the quality of care.^42,44^

Administrative and organisational structures in constant change were often routine in nurses' practice; however, these situations contributed to negative outcomes such as dissatisfaction, burnout, and high levels of human resource turnover, which not only influenced nurses' performance but also seriously affected the quality of care and patient outcomes, with special emphasis on patient safety.^33,35,40,41^

### Professional development opportunities

The promotion of professional development was an essential feature of a positive nursing practice environment.^40,41^ The qualification of professionals and the empowerment of the team to promote the safety and quality of care was essential.^39,42^ The negative perception of the 'continuing education' domain found in Souza’s study^33,35^ may have consequences for patient safety, since the meeting between training and clinical practice, the updating and recycling of knowledge, and the development of critical thinking about practices are essential for the promotion of patient safety.^33,35^

### Strategies to promote patient safety and positive nursing practice environments

Healthcare organisations have a key role in promoting positive and safer nursing practice environments for patients. Institutions should be proactive in promoting the health and wellbeing of health professionals, with clear consequences such as higher levels of team performance, better patient care, higher levels of staff retention, and decreased absences owing to illness.^39,42^

The following strategies to improve work environments and patient safety were mentioned in the studies that were included in this review: the development of patient safety protocols specific to the primary healthcare setting; continuing education and training of professionals; improvement of working conditions and infrastructure; improvement of communication; prevention of errors^33,35^ and implementation of incident reporting systems; development of scientific evidence-based practice as a basis for decision-making; involvement of patients and family in care; and sensibilisation of professionals and managers for the practice of safe care.^32,34^

Clinical supervision sessions, as a process of monitoring nurses' practice, also emerged as a strategy that promotes self-reflection and open communication, with clear results in improving professional satisfaction and wellbeing of professionals, which can be reflected in higher-quality and safer care.^40,43^

Implementing planned break areas designed to provide staff with a place where they can retreat from the clinical environment to reflect or gain comfort provided a positive atmosphere with opportunities for communication between professionals, and where they could find advice and reinforcement from their peers.^40,43^ The existence of personal space, privacy, and quiet spaces in the work environment could also have a beneficial effect on nurses' health, with consequences for their performance and desire to remain in the nursing profession.^42,44^ The mental health and wellbeing of healthcare professionals should be carefully supervised and evaluated. Promoting mental health and wellbeing by implementing supportive measures for professionals produced many benefits, such as reducing stress and improving the quality of care.^40,43^

## Discussion

### Summary

The initial purpose of this study was to understand the impact that nursing practice environments have on patient safety culture in the specific context of primary health care. Although detailed evidence was not found, it was clear how various characteristics of nursing practice environments can impact the safety of care, which in addition to proving the originality of this study, translates into the need to maintain this line of research. Despite the differences between countries, the present study found that there is a well-defined body of knowledge that associates favourable nursing practice environments with better quality and safer care.^37,39,40,42,44^ The association between nursing practice environments, and their components, and patient safety-related outcomes has been explored over the years, although with more emphasis on the hospital setting.^4,5,37,43,44,54–58^ However, several characteristics of nursing practice environments that influence patient outcomes, such as quality of care and patient safety, can be extrapolated to other settings such as primary health care. Additionally, the multifactorial dimension of nursing practice environments was found, illustrating that positive nursing practice environments are linked not only to the availability of a sufficient number of human resources but also to organisational culture and leadership, both of which are heavily influenced by organisational policies.^4,39,42^

### Strengths and limitations

This scoping review used a rigorous and transparent method, guided by a protocol reviewed by an experienced research team, which included an expert in producing scientific evidence according to the JBI method. The extensive literature search conducted, which included five grey literature databases, the pilot study to assess eligibility criteria, and the review by two independent reviewers, are strengths of the review. Regarding the limitations of this review, many of the included studies examined the perspective of different primary healthcare professionals, which may have skewed the findings because the identified issues may not necessarily apply to nurses, despite the assumption that they may interfere with nurses' practice. This review is also limited by the fact that most of the research done in recent years has been done in hospitals. As a result, the authors believe that primary healthcare research should be encouraged because it may bring valuable contributions and serve as a foundation for the definition and implementation of strategies to improve nursing practice environments and care safety.

### Comparison with existing literature

The existence of unfavourable nursing practice environments affects nursing practice, making nurses unable to effectively use their skills and knowledge to provide quality care and ensure patient safety.^37,40^ On the other hand, positive nursing practice environments allow nurses to use their advanced training for higher-quality and safer care.^40^ There is also an association that nurses with greater training have greater perceived autonomy, and can establish more effective collaborations with professionals from other disciplines. This in turn is associated with a reduction in adverse events such as urinary tract infections, pneumonia, cardiac arrest, and reduced length of stay.^38,58^

Relationships among nurses and between nurses, and other members of the multidisciplinary team, are equally important factors that influence the attributes of nursing practice environments.^32,41,57,58^ Problems related to collegial relationships between professionals and communication difficulties are associated with increased reporting of medication errors.^18,56,57^ It is understood that collaborative care with other professionals and communication skills are factors that promote more supportive professional practice environments with better safety culture scores.^18,37,44,57^

Stress has emerged in the literature as a recurring element in research, and there is great concern about stress in healthcare professionals in general.^37,39,40,42,43^ Stressful work environments are known to be more propitious to the existence of errors, and to the promotion of unsafe and lower-quality care.^34,37,42,44^

The entire team must participate in this change to make it more effective, and the adoption of patient safety protocols that involve patients and their families as well as professional continuing education is seen as essential for the development of institutional safety culture.^17,34^ Mesquita discovered that managers were committed to patient safety despite the negative evaluation of the patient safety culture, confirming the existence of several factors that influence patient safety.^34^ The same was found in other studies, which showed that while managerial support is important, other things such as heavy workloads or a lack of communication have a big negative effect on nurses and may make patient care less safe.^4^

Nurse managers and healthcare institutions' administrations have a leading role, either in the design of measures to support nursing practice environments to be conducive to the promotion of safer care, or by involving professionals in institutional policies, promoting their job satisfaction, and reducing professional turnover.^37,40,41,44,57,59^ The role of nurse leaders in empowering staff through easier access to support, resources, and information is essential to promote work engagement, professional autonomy, and nurses' physical, psychological, and emotional wellbeing, which in turn may increase nurses' positive attitudes and skills needed to provide safer care.^4,37,57^ Improved job satisfaction, organisational commitment,^3^ and patient-related outcomes can be achieved through supportive work environments, active leadership in nursing, and leader empowerment behaviours, which will improve the safety culture.^59^

### Implications for practice

Nursing staff constitute the majority of healthcare professionals and are the main contributors to patient outcomes.^7,18^ The American Nurses Association (ANA) even refers to patient outcomes as sensitive to nursing care, since most of them can be directly affected by the care provided by nurses.^60^ This denotes how important nursing is to health systems and how important it is to find out what influences this workforce. The issue of the shortage of nurses that the WHO^61^ has cautioned about, the high levels of stress and burnout, and the turnover of nurses and the consequences of fatigue show the huge urgency to implement interventions that improve the working conditions of nurses.^42,44^

Significant contributions to adverse event prevention can be made by understanding professional attitudes,^32^ which is essential information for nurse managers, for improving knowledge about primary healthcare nurses, and the subsequent and necessary definition of strategies that promote positive nursing practice environments. Primary healthcare error and adverse event reduction is a global priority that has a significant impact on population health care.

## Review Registration

Open Science Framework: https://osf.io/wy48r ; https://doi.org/10.17605/OSF.IO/JG3HE.

## Data

The dataset relied on in this article is available from the corresponding author on reasonable request.

## References

[1] Malinowska-Lipień I, Micek A, Gabryś T (2021). Impact of the work environment on patients’ safety as perceived by nurses in Poland—a cross-sectional study. Int J Environ Res Public Health.

[2] World Health Organization (WHO) (2009). Conceptual framework for the International classification for patient safety. Version 1.1: final technical report.

[3] WHO (2020). World patient safety day, 17 September 2020. Charter — Health worker safety: a priority for patient safety.

[4] Mihdawi M, Al-Amer R, Darwish R (2020). The influence of nursing work environment on patient safety. Workplace Health Saf.

[5] International Council of Nurses (ICN) (2007). [Positive practice environments: quality workplaces = quality patient care: information and action tool kit] Ambientes Favoráveis À Prática: Condições de Trabalho = Cuidados de Qualidade: Instrumentos de Informação E Acção (in Portuguese) (Geneva, ICN).

[6] Health and Safety Executive (2007). Managing the causes of work-related stress: a step by step approach using the Management Standards.

[7] Institute of Medicine (2004). Keeping patients safe: transforming the work environment of nurses.

[8] Jarrar M, Al-Bsheish M, Aldhmadi BK (2021). Effect of practice environment on nurse reported quality and patient safety: the mediation role of person-centeredness. Healthcare (Basel).

[9] Pimenta Lopes Ribeiro OM, de Lima Trindade L, Silva Fassarella C (2022). Impact of COVID-19 on professional nursing practice environments and patient safety culture. J Nurs Manag.

[10] Paranaguá T de B, Bezerra ALQ, Tobias GC, Ciosak SI (2016). Support for learning in the perspective of patient safety in primary health care. Rev Lat Am Enfermagem.

[11] Silva A da, Backes DS, Magnago T de S, Colomé JS (2019). Patient safety in primary care: conceptions of family health strategy nurses. Rev Gaucha Enferm.

[12] Ribeiro H, Pardini R, Silva J, Menezes A (2021). [Patient safety in primary health care: the perceptions of professionals working in family health teams] *Segurança do Doente NA Atenção Primária: Perceção de Profissionais de Equipas de Saúde DA Família* (available in Portuguese and English). Revista de Enfermagem Referência.

[13] WHO and the United Nations Children’s Fund (UNICEF) (2018). A vision for primary health care in the 21st century: towards universal health coverage and the sustainable development goals.

[14] WHO and UNICEF (2020). Operational framework for primary health care: transforming vision into action.

[15] El Shafei AMH, Zayed MA (2019). Patient safety attitude in primary health care settings in Giza, Egypt: cross-sectional study. Int J Health Plann Manage.

[16] Schwappach DLB, Gehring K, Battaglia M (2012). Threats to patient safety in the primary care office: concerns of physicians and nurses. Swiss Med Wkly.

[17] Kuriakose R, Aggarwal A, Sohi RK (2020). Patient safety in primary and outpatient health care. J Family Med Prim Care.

[18] Lawati MHA, Dennis S, Short SD, Abdulhadi NN (2018). Patient safety and safety culture in primary health care: a systematic review. BMC Fam Pract.

[19] Tabrizchi N, Sedaghat M (2012). The first study of patient safety culture in Iranian primary health centers. Acta Med Iran.

[20] Webair HH, Al-Assani SS, Al-Haddad RH (2015). Assessment of patient safety culture in primary care setting, Al-Mukala, Yemen. BMC Fam Pract.

[21] Ghobashi MM, Ghani El-ragehy HA, Mosleh H, Al-Doseri FA (2014). Assessment of patient safety culture in primary health care settings in Kuwait. Epidemiol Biostat Public Health.

[22] Lucas P, Nunes E (2020). Nursing practice environment in primary health care: a scoping review. Rev Bras Enferm.

[23] Nora CRD, Beghetto MG (2020). Patient safety challenges in primary health care: a scoping review. Rev Bras Enferm.

[24] Michel P, Brami J, Chanelière M (2017). Patient safety incidents are common in primary care: a national prospective active incident reporting survey. PLoS One.

[25] Khalil H, Bennett M, Godfrey C (2020). Evaluation of the JBI scoping reviews methodology by current users. Int J Evid Based Healthc.

[26] Peters MDJ, Godfrey CM, Khalil H (2015). Guidance for conducting systematic scoping reviews. Int J Evid Based Healthc.

[27] Tricco AC, Lillie E, Zarin W (2018). PRISMA extension for Scoping reviews (PRISMA-SCR): checklist and explanation. Ann Intern Med.

[28] Pereira S de A, Ribeiro O, Fassarella CS, Santos EJF (2023). The impact of nursing practice environments on patient safety culture in primary health care: a scoping review protocol. BJGP Open.

[29] Peters MDJ, Godfrey C, McInerney P, Aromataris E, Munn Z (2020). JBI Manual for Evidence Synthesis.

[30] Page MJ, McKenzie JE, Bossuyt PM (2021). The PRISMA 2020 statement: an updated guideline for reporting systematic reviews. BMJ.

[31] Bardin L (2011). Content analysis (5th ed).

[32] Gabrani JC, Knibb W, Petrela E (2016). Provider perspectives on safety in primary care in Albania. J Nurs Scholarsh.

[33] Souza MM, Ongaro JD, Lanes TC (2019). Patient safety culture in the primary health care. Rev Bras Enferm.

[34] Mesquita KO (2017). [Culture of patient safety in primary health care] Cultura de segurança do paciente na atenção primária à saúde (in Portuguese) [dissertation]. Brazil: Federal University of Ceará.

[35] Souza MM (2017). [Patient safety culture in the Primary Health Care] Cultura de segurança do paciente na atenção primária à saúde (in Portuguese) [dissertation]. Brazil, Federal University of Santa de Santa Maria.

[36] Coetzee SK, Klopper HC, Ellis SM, Aiken LH (2013). A tale of two systems—nurses practice environment, well being, perceived quality of care and patient safety in private and public hospitals in South Africa: a questionnaire survey. Int J Nurs Stud.

[bib37] Alharbi AA (2018). The impact of nurse work environment on nurse outcomes, nurse-perceived quality of care and patiente safety in Saudi Arabia [dissertation].

[38] Richardson A, Storr J (2010). Patient safety: a literature [corrected] review on the impact of nursing empowerment, leadership and collaboration. Int Nurs Rev.

[39] Schalk DM, Bijl ML, Halfens RJ (2010). Interventions aimed at improving the nursing work environment: a systematic review. Implement Sci.

[40] Poghosyan L, Boyd DR, Clarke SP (2016). Optimizing full scope of practice for nurse practitioners in primary care: a proposed conceptual model. Nurs Outlook.

[41] Stalpers D, de Brouwer BJM, Kaljouw MJ, Schuurmans MJ (2015). Associations between characteristics of the nurse work environment and five nurse-sensitive patient outcomes in hospitals: a systematic review of literature. Int J Nurs Stud.

[42] Burke M (2013). Managing work-related stress in the district nursing workplace. Br J Community Nurs.

[43] Rogers A (2021). Promoting health and wellbeing across community nursing teams: role of the specialist practitioner district nurse. Br J Community Nurs.

[44] Nejati A, Shepley M, Rodiek S (2016). A review of design and policy interventions to promote nurses' restorative breaks in health care workplaces. Workplace Health Saf.

[45] West M, Bailey S, Williams E (2020). The courage of compassion: supporting nurses and midwives to deliver high-quality care.

[bib46] McKinless E (2020). Impact of stress on nurses working in the district nursing service. Br J Community Nurs.

[47] Storey C, Cheater F, Ford J, Leese B (2009). Retaining older nurses in primary care and the community. J Adv Nurs.

[48] Han K, Trinkoff AM, Geiger-Brown J (2014). Factors associated with work-related fatigue and recovery in hospital nurses working 12-hour shifts. Workplace Health Saf.

[bib49] Mudallal RH, Othman WM, Al Hassan NF (2017). Nurses' burnout: the influence of leader empowering behaviors, work conditions, and demographic traits. Inquiry.

[50] Dall’Ora C, Ball J, Reinius M, Griffiths P (2020). Burnout in nursing: a theoretical review. Hum Resour Health.

[51] Dall’Ora C, Griffiths P, Ball J (2015). Association of 12 H shifts and nurses' job satisfaction, burnout and intention to leave: findings from a cross-sectional study of 12 European countries. BMJ Open.

[52] Chemali S, Mari-Sáez A, El Bcheraoui C, Weishaar H (2022). Health care workers' experiences during the COVID-19 pandemic: a scoping review. Hum Resour Health.

[53] Majrabi M (2022). Nurses burnout, resilience and its association with safety culture: a cross sectional study. Open J Nurs.

[54] Aiken LH, Sermeus W, Van den Heede K (2012). Patient safety, satisfaction, and quality of hospital care: cross sectional surveys of nurses and patients in 12 countries in Europe and the United States. BMJ.

[55] You L, Aiken LH, Sloane DM (2013). Hospital nursing, care quality, and patient satisfaction: cross-sectional surveys of nurses and patients in hospitals in China and Europe. Int J Nurs Stud.

[56] Lake ET, Hallowell SG, Kutney-Lee A (2016). Higher quality of care and patient safety associated with better NICU work environments. J Nurs Care Qual.

[57] Van Bogaert P, Timmermans O, Weeks SM (2014). Nursing unit teams matter: impact of unit-level nurse practice environment, nurse work characteristics, and burnout on nurse reported job outcomes, and quality of care, and patient adverse events—a cross-sectional survey. Int J Nurs Stud.

[58] Papastavrou E, Efstathiou G, Lemonidou C (2014). Cypriot and Greek nurses' perceptions of the professional practice environment. Int Nurs Rev.

[59] Zraychikova E, Gehri B, Zúñiga F (2023). The interaction between leadership, the patient-to-nurse ratio and nurses' work-life balance in the psychiatric inpatient setting in Switzerland: a secondary data analysis of cross-sectional data. Adm Policy Ment Health.

[60] Montalvo I (2007). The National database of nursing quality indicators™ (NDNQI®). Online J Issues Nurs.

[61] WHO (2010). A brief Synopsis on patient safety.

